# Medical Items

**Published:** 1892-03

**Authors:** 


### Medical Items.

The Forty-third Annual Session of the Medical Association of Georgia will meet in Columbus, Ga., on April 20, 21, 22.

The officers are: President, G. W. Mulligan, M. D., of Washington, Ga.; Vice-Presidents, James M. Hull, M. D., of Augusta, Mark H. O'Daniel, M. D., of Macon; Treasurer, E. C. Goodrich, M. D., of Augusta; Secretary, Dan. H. Howell, M. D., of Atlanta.

---

Mr. W. B. Saunders, 913 Walnut street, Philadelphia, announces that he has in press two important text-books, which, we doubt not, will be given a cordial reception by American readers. The one is "An American Text-book of Surgery," by Professors Keen, White and others. "An American Text-book of the Theory and Practice of Medicine," edited by Professor William Pepper, is the other. Among the contributors to these two works are the best known teachers in American surgery and medicine.

---

The Trustees and Faculty of the Southern Medical College, this city, are preparing for a new college building. The new college will probably be situated in the neighborhood of the Grady Hospital, and it is hoped, will be ready for occupancy by the opening of the fall session.

The Southern Medical Society, an undergraduate medical society of the Southern Medical College gave a banquet to the Faculty of the college and members of the graduating class at Vignaux' restaurant, on the evening of March 3d. Our space does not permit us to publish the list of toasts with the appropriate quotations appended thereto, but will only say, using one of them, that

> So, many a tongue the evening hour prolonged,
> With spangled speeches.

---

Dr. B. W. Bizzell, who formerly practiced medicine in Atlanta and then moved to Mississippi, has returned to this city to resume his labors.

EASTERN ASSURANCE AND WESTERN PLAGIARISM.—An editorial paragraph, in the February *Western Medical Reporter*, Chicago, headed "Eastern Assurance" says:

The *Medical Record* now and again takes a slap at Western medicine. Recently, the *Record* insinuated that Chicago had no medical journals. Chicago has also no men who appropriate the work of Eastern men and call it their own. The latest monumental appropriation is Wyeth's operation of bloodless amputation at the hip joint. This operation was presented by Dr. Muscroft, Cincinnati, Ohio, to the Cincinnati Academy of Medicine, March 14, 1887, and published in the *Lancet Clinic* April 2, 1887. New York might not be so pompous if she gave honor to whom honor is due. Dr. Shrady is welcome to this bit of information as he aspires to be a live journalist.

Whereupon we rise to observe that unfortunately the same issue of the *Reporter* contains an article by an Illinois physician, on "What I Learned to Unlearn in Gynecology," which, in point of experience described and diction employed, is almost an exact reproduction of an article by Dr. William Goodell, of Philadelphia, on the same subject in the *Medical Record* of last year. We commend the above paragraph, and the article in question, which was published without the author's permission with the name of the Illinois gynecologist attached, to the indulgent consideration of Dr. Goodell and Dr. Shrady, the distinguished editor of the *Record*. Curious and funny, isn't it?

---

TREATMENT OF SOFT CHANCRE.—Drop peroxide solution upon the surface until the pus is removed and bubbling ceases. Bathing with warm water before making the application is advisable. This can be done morning and evening, and a simple water dressing applied upon absorbent cotton, or black wash used where a stimulant effect is desired. If a dry dressing is desired the following formula:

℞.—Hydrarg. chlor. mitis, ʒj.

Bismuth. subnitrat.,

Pulv. cinchonæ flavæ, aa ʒij. M.

has proved very efficient and is recommended by Dr. Davis, of Bridgeton, N. Y.—*Dietetic Gazette*.

